# Development and maintenance of the brain's immune toolkit: Microglia and non‐parenchymal brain macrophages

**DOI:** 10.1002/dneu.22545

**Published:** 2017-10-24

**Authors:** Jose P. Lopez‐Atalaya, Katharine E. Askew, Amanda Sierra, Diego Gomez‐Nicola

**Affiliations:** ^1^ Instituto de Neurociencias de Alicante, Universidad Miguel Hernández‐Consejo Superior de Investigaciones Científicas (UMH‐CSIC), Avenida Ramón y Cajal, s/n, Sant Joan d'Alacant Spain; ^2^ Southampton General Hospital, Biological Sciences, University of Southampton, South Lab&Path Block, LD80C, MP840 SO166YD Southampton United Kingdom; ^3^ Achucarro Basque Center for Neuroscience Leioa 48940 Spain; ^4^ Ikerbasque Foundation Bilbao 48013 Spain; ^5^ University of the Basque Country EHU/UPV Leioa 48940 Spain

**Keywords:** microglia, development, lineage, progenitor, self‐renewal

## Abstract

Microglia and non‐parenchymal macrophages located in the perivascular space, the meninges and the choroid plexus are independent immune populations that play vital roles in brain development, homeostasis, and tissue healing. Resident macrophages account for a significant proportion of cells in the brain and their density remains stable throughout the lifespan thanks to constant turnover. Microglia develop from yolk sac progenitors, later evolving through intermediate progenitors in a fine‐tuned process in which intrinsic factors and external stimuli combine to progressively sculpt their cell type‐specific transcriptional profiles. Recent evidence demonstrates that non‐parenchymal macrophages are also generated during early embryonic development. In recent years, the development of powerful fate mapping approaches combined with novel genomic and transcriptomic methodologies have greatly expanded our understanding of how brain macrophages develop and acquire specialized functions, and how cell population dynamics are regulated. Here, we review the transcription factors, epigenetic remodeling, and signaling pathways orchestrating the embryonic development of microglia and non‐parenchymal macrophages. Next, we describe the dynamics of the macrophage populations of the brain and discuss the role of progenitor cells, to gain a better understanding of their functions in the healthy and diseased brain. © 2017 Wiley Periodicals, Inc. Develop Neurobiol 78: 561–579, 2018

## INTRODUCTION

**Figure 1:**
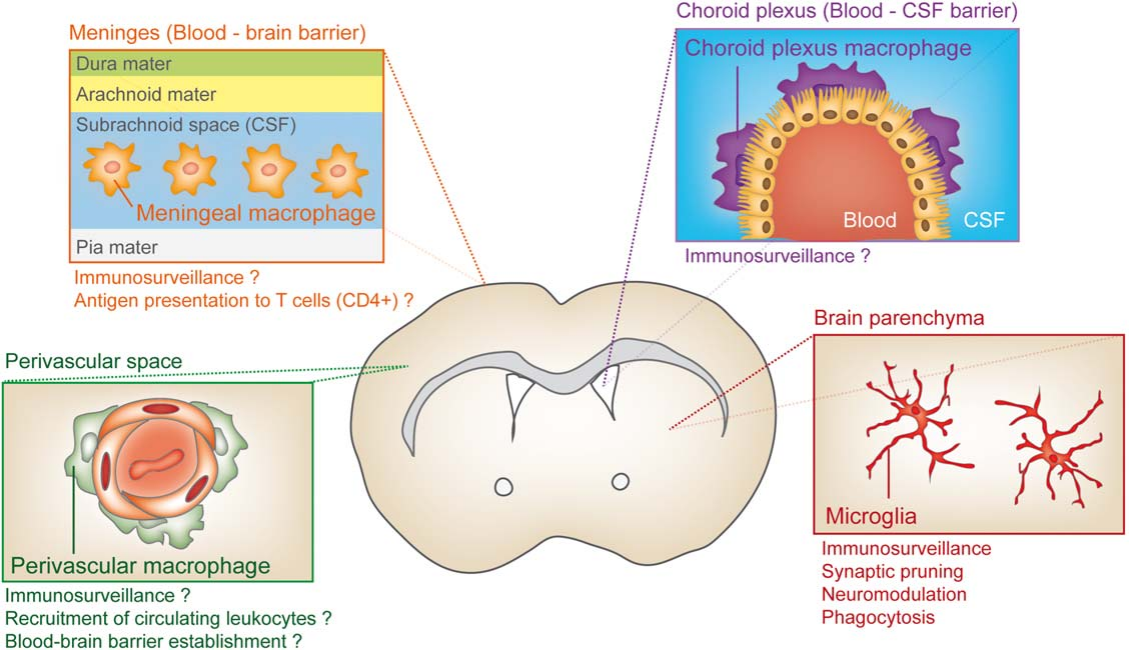
Diversity of myeloid cell types in the adult CNS. The CNS is filled with a variety of resident innate immune cells that regulate homeostasis and execute surveillance tasks. Microglia cells tile the entire brain in a contiguous and essentially non‐overlapping manner that is orderly and well organized to actively screen the brain parenchyma for incoming threats. Three other major types of brain‐resident macrophages are present in the outer boundaries of the brain, such as the perivascular space, choroid plexus, and in the meninges where it is thought they constitute the first line of host defense against cellular or pathogenic components. [Color figure can be viewed at http://wileyonlinelibrary.com]

Microglia and brain macrophages are myeloid linage cells strategically located throughout the brain parenchyma and barrier regions (i.e., perivascular space, meninges, and choroid plexus), where they ingest and degrade dead cells, debris, and foreign material and orchestrate inflammatory processes (Ransohoff & Cardona, 2010) (Fig. 1). The study of their specialized functions and the dynamics of these distinct populations should contribute to advance our current knowledge about their role in disease, and may open new avenues for the development of novel targeted therapies. In this review, we will discuss the mechanisms governing commitment of primitive myeloid progenitors to a tissue‐specific macrophage fate, with a focus on brain‐resident macrophages, and elaborate on the dynamics and functions of these distinct populations.

## EMBRYONIC ORIGINS OF THE BRAIN‐RESIDENT MACROPHAGES

Microglia and other resident macrophages of the central nervous system (CNS), including perivascular, meningeal, and choroid plexus macrophages, originate during primitive hematopoiesis from prenatal erythromyeloid precursors (EMPs) found in the yolk sac (Schulz et al., 2012; Hashimoto et al., 2013; Kierdorf et al., 2013a; Gomez Perdiguero et al., 2015; Hoeffel et al., 2015; Sheng et al., 2015; Goldmann et al., 2016). All brain macrophages, with the exception of choroid plexus macrophages, are maintained locally throughout adulthood by self‐renewal (Goldmann et al., 2016; Askew et al., 2017; Reu et al., 2017; Tay et al., 2017) (discussed in greater detail in “Macrophage population dynamics in the adult brain” Section). Embryonic hematopoiesis starts at the yolk sac around embryonic day (E) 7.5 and microglial precursors cells reach the neuroepithelium by E9.5–10 (Alliot et al., 1999; Ginhoux et al., 2010). Immunophenotyping analyses have revealed that uncommitted EMPs express specific markers such as CD31^+^ and c‐Kit^+^ (Kierdorf et al., 2013a). EMPs develop via the macrophage ancestor population A1 (CD45^+^, CX3CR1^low^, F4/80^low^) into the A2 (CD45^+^, CX3CR1^hi^, F4/80^hi^) progenitor population that commit to microglial cells (Kierdorf et al., 2013a). Similar observations were made in tamoxifen‐dependent Cre transgenic mouse lines under the control of the colony stimulating factor‐1(CSF‐1R) or Runx1 promoters, where Ginhoux and colleagues further uncovered the existence of two waves of temporally separated and functionally distinct EMPs that emerge in the yolk sac between E7.5 and E8.5. The first of these waves emerges from E7.5 and consist of CSF‐1R^hi^, c‐Myb^‐^ EMPs that give rise to yolk sac macrophages that will colonize the embryonic brain rudiments to generate microglia (Hoeffel et al., 2015; Hoeffel & Ginhoux, 2015; Ginhoux & Guilliams, 2016).

In contrast to the unequivocally established origin of microglial cells, the ontogeny of non‐parenchymal macrophages has remained less clear. Whereas until recently, macrophages at brain interfaces were tought to mainly develop postnatally from short‐living blood monocytes that are quickly replaced by bone marrow‐derived cells, new evidence using powerful genetic fate‐mapping approaches and single‐cell transcriptomic profiling, indicates that non‐parenchymal macrophages share a common ontogenetic origin with microglia but still represent a distinct specialized populations of tissue macrophages (Goldmann et al., 2016). Loss of function experiments in knockout mice further demonstrated that the development of microglia and macrophages at brain interfaces is independent of the master transcription factor Myb, but largely depends on transcription factors like Runx1, Pu.1, and interferon regulatory factor 8 (Irf8) (Kierdorf et al., 2013a; Goldmann et al., 2016). New efforts are granted to further expand our knowledge on the embryonic hematopoietic niche where primitive progenitors expand and differentiate during development to generate the distinct populations of non‐parenchymal macrophages of the brain. The generation of novel genetic tools with high temporal resolution will allow for a better understanding of the ontogeny of tissue‐resident macrophages including macrophages at brain interfaces.

## SPECIFICATION OF BRAIN‐RESIDENT MACROPHAGES DURING DEVELOPMENT

**Figure 2:**
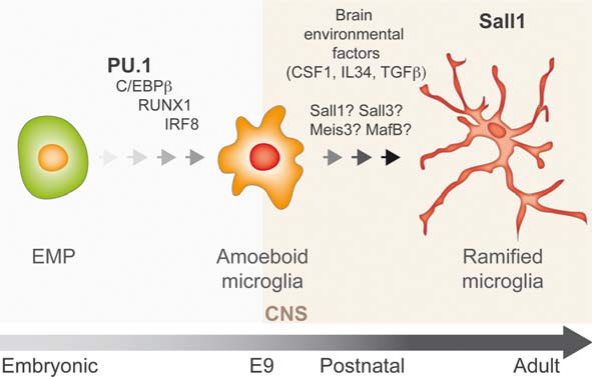
From EMP to microglia. Microglia arise early during development from EMP in the embryonic yolk sac that seed the mouse brain rudiment around E9.0 upon commitment to immature macrophage cells showing ameboid morphology and a high proliferation rate (also known as A cells; Bertrand et al., 2005). Amoeboid cells persist during the first 2 weeks of the postnatal brain where they gradually acquire the ramified shape characteristic of the active microglia in the steady state. Commitment of EMP through immature ameboid macrophages, towards differentiate microglia is regulated by intrinsic genetic programs and environmental signals. Initially, a small subset of master regulators of macrophage development, including, PU.1, C/EBPs, RUNX1, and IRF8, cooperatively drives specification and fate acquisition of EMPs into immature macrophages. In the brain, environmental factors such as CSF1, IL34, and TGFβ play fundamental roles in shaping, maintaining, and reinforcing microglial identity. Recent genome‐wide analyses have identified several transcription factors that are specific or highly enriched in microglia. These include SALL1, SALL3, MEIS3, and MAFB. However, their roles in microglia biology remain to be elucidated. To date, SALL1 have been shown to maintain microglia identity and to regulate phenotypic plasticity. [Color figure can be viewed at http://wileyonlinelibrary.com]

In recent years, the development of fate mapping approaches combined with next‐generation sequencing and, more recently, the advent of single‐cell genomics, has led to a crucial turning point in our understanding of how brain's resident macrophages develop and acquire specialized functions (Prinz et al., 2017). Microglia and non‐parenchymal brain macrophages (meningeal, perivascular, and choroid plexus macrophages) develop from precursor cells that evolve through intermediate progenitors in a fine‐tuned process in which intrinsic factors and external stimuli combine to progressively sculpt their genome architecture through epigenetic mechanisms leading to cell type‐specific transcriptional profiles (Crotti and Ransohoff, 2016; Prinz et al., 2017) (Fig. 2).

### Master Regulators of the Fate and Differentiation of Brain Macrophages

During the developmental journey from progenitors to brain macrophages, a relatively small number of transcription factors, including RUNX1 (Runt‐related transcription factor 1), PU.1, and IRF8, orchestrate lineage commitment of yolk sac myeloid precursors in brain macrophages (Prinz & Priller, 2014; Prinz et al., 2017). These transcription factors act in a combinatorial manner to promote the acquisition of cell fate and the maintenance of cellular identity (Heinz et al., 2015).

*Runx1* is expressed in the hematopoietic precursors of the yolk sac (North et al., 1999; Samokhvalov et al., 2007) where it is a direct target of the master regulator of hematopoiesis SCL/TAL1 (Stem cell leukemia/T‐cell acute lymphoblastic leukemia 1) (Landry et al., 2008). The *Runx1* gene locus has been critical in cell‐tracing experiments to demonstrate that parenchymal brain macrophages arise from primitive myeloid progenitors originated from extra‐embryonic yolk sac (Ginhoux et al., 2010; Zusso et al., 2012). Similar to observations in other cell types of the myeloid lineage, RUNX1 regulates proliferation of microglial cells and differentiation to the ramified morphology typically observed in the adult forebrain (Zusso et al., 2012). A new experimental study carried out in mice and humans shows that RUNX1‐binding motif is enriched at the enhancer landscape of adult mouse and human microglia cells (Gosselin et al., 2017). Another master transcription factor for microglia and macrophage development is PU.1, a myeloid lineage‐determining factor that belongs to Class III ETS family of transcription factors (Klemsz et al., 1990; Wei et al., 2010). *Pu.1* is a major downstream target gene of RUNX1 during embryonic haematopoiesis (Huang et al., 2008). Mice lacking PU.1 show complete absence of microglia and other CNS macrophages, without affecting the stem cell compartment (c‐Kit^+^ EMP cells) (Beers et al., 2006; Kierdorf et al., 2013a; Goldmann et al., 2016). In Zebrafish, during embryonic myelopoiesis, *Pu.1* and *Runx1* are regulated by a negative feedback loop that governs cell commitment between distinct myeloid fates (Jin et al., 2012). The third major critical transcription factor playing critical roles in cell‐fate decisions of myeloid cells is IRF8 (Holtschke et al., 1996). Early studies on myeloid differentiation in the adult hematopoietic system demonstrated that IRF8 regulates the acquisition monocytic/macrophage fate (Scheller et al., 1999; Tamura et al., 2000; Hambleton et al., 2011). *Irf8* knockout in mice and zebrafish results in impaired microglia development (Kierdorf et al., 2013a; Shiau et al., 2015). Prinz and colleagues have shown that IRF8 can act both independently and as heterodimeric partner of PU.1 to regulate the differentiation of microglia from yolk sac precursors (Kierdorf et al., 2013a). Whereas *Pu.1* knockout mice are devoid of microglia, *Irf8*‐ablated mice show an overall reduction of microglia density (Kierdorf et al., 2013a). *Irf8* knockout yolk sac show a dramatic reduction in EMP‐derived macrophage ancestor population A2, but preserved levels of A1 cells, suggesting a role of IRF8 in maturation of intermediate progenitors toward mature microglia (Kierdorf et al., 2013a). Interestingly, the few remaining A2 cells in *Irf8* knockout mice can still proliferate and give rise to a microglia population in the adult that is only slightly decreased as compared with wild‐type mice (Kierdorf et al., 2013a). Parenchymal macrophages in the adult brain of *Irf8*‐deficient mice display reduced ramification and lower morphological complexity, and altered levels of microglial markers such as Iba1 (Minten et al., 2012). In line with these data, a recently published transcriptome‐based profiling of yolk sac‐derived macrophages has shown a critical role of IRF8 on the maturation throughout development and adulthood of microglia and other types of tissue‐restricted macrophages (Hagemeyer et al., 2016). IRF8 is also critical for the development of meningeal macrophages whereas choroid plexus macrophages develop normally under IRF8 deficiency (Goldmann et al., 2016). In addition to these master regulators, recent genomic experiments have suggested a role for the transcription factors SALL1 (Spalt like transcription factor 1), SALL3 (Spalt like transcription factor 3), MEIS3 (Meis homeobox 3), and MAFB (MAF BZIP transcription factor B) in development and function of developing and adult microglia (Mass et al., 2016, Matcovitch‐Natan et al., 2016; Gosselin et al., 2017). So far, loss of function experiments has clearly established a role for SALL1 in the regulation of microglia phenotype and function (Buttgereit et al., 2016). Buttgereit et al. (2016) found that SALL1 is expressed exclusively in microglia and that microglia‐specific deletion of Sall1 triggers phenotypic transition to an inflammatory reactive state. These data strongly suggest that, under physiological conditions, SALL1 is actively repressing a transcriptional gene program that maintains microglia steady‐state. Although the progress in the field has been significant in the last years, a better understanding of the regulatory program that controls development of microglia is needed. Future research will further elucidate the nuances promoting precise control of microglial fate by master regulators. Only very recently we have begun to understand the development and differentiation of non‐parenchymal brain macrophages, and much work remains to be done to understand their exact nature and origin.

### Enhancer Selection Drives Acquisition and Maintenance of Tissue‐Resident Macrophage Identity

Differentiation from progenitor and intermediate cell types to fully differentiated brain macrophages requires the timely control of gene expression and this is intimately linked with epigenetic mechanisms. In the genome, distant acting *cis*‐regulatory sequences or enhancers are bound by transcription factors and co‐activators to enhance the transcription of an associated gene. Enhancers are structurally and functionally defined by presence of specific post‐translational modifications on the core histone tails of the chromatin. Monomethylation of H3K4 (H3K4me1), and the concomitant presence of H3K27 acetylation discriminate strong from weak enhancers (H3K27ac) (Heintzman et al., 2009, 2007; Creyghton et al., 2010). Increased DNA accessibility and CBP/p300 binding are other distinctive features of active enhancers (Kim et al., 2010; Visel et al., 2013) Tissue‐ and cell type‐specific signatures of active enhancers were identified by genomic studies comparing many tissues and cell types (Shen et al., 2012, Thurman et al., 2012). Moreover, evidence supports that enhancers and super‐enhancers—large clusters of enhancers—play key roles in the acquisition and maintenance of cell identity (Rada‐Iglesias et al., 2011; Hnisz et al., 2013; Whyte et al., 2013; Adam et al., 2015). With approximately 12,000 active promoters, mouse macrophages contain between 35,000 and 45,000 epigenetically marked enhancer regions (Ghisletti et al., 2010; Heinz et al., 2010; Kaikkonen et al., 2013). Although all cell types express hundreds of transcription factors, a large fraction of functional enhancers are characterized by the presence of a relatively small set of lineage determining transcription factors. In the case of macrophages, these include PU.1, AP‐1, and C/EBPs (Ghisletti et al., 2010; Heinz et al., 2010). Seminal studies comparing peritoneal macrophages and splenic B cells have shown that macrophage specific *cis*‐regulatory elements are bound by the pioneer transcription factor PU.1, which facilitates the subsequent binding of other lineage‐determining factors including C/EBPs, IRF8, and AP‐1 at adjacent locations, and the deposition of H3K4me1, to confer transcriptional function to cell type‐specific distal regulatory elements (Heinz et al., 2010). Using wisely designed experiments relying on naturally occurring genetic variation between different inbred mouse strains as an “*in vivo* mutagenesis screen”, they found that polymorphisms at strain‐specific PU.1‐bound enhancers were highly enriched in comparison with strain‐similar PU.1‐bound enhancers (Heinz et al., 2013). Together, these data strongly suggest a hierarchical model, in which macrophage‐specific enhancer selection by PU.1 required collaborative interactions with additional macrophage‐restricted lineage‐determining transcription factors (Heinz et al., 2010, 2013). Recent evidence indicates that PU.1‐bound sites in the genome of human and mouse microglia are largely conserved and correspond to genomic regions of open chromatin associated with methylated histones H3K4me2 and H3K27ac. Moreover, these regulatory regions were found to be enriched in motifs for IRF, AP‐1, MEF2, C/EBP, and RUNX (Gosselin et al., 2017). This study extends previous findings in peritoneal macrophages to mouse and human microglia and provide novel insights on the fundamental role of PU.1 in the establishment of the enhancer landscape of microglia cells (Gosselin et al., 2017). Again, very little is known about the genomic landscape of *cis*‐regulatory elements present in other types of brain macrophages. The identification of the specific and shared enhancers across the different populations of brain macrophages will greatly contribute to our understanding on their specific functions in the healthy and diseased CNS.

### miRNA Control of Brain Macrophage Differentiation and Function

A recently discovered factor controlling the epigenetic landscape is microRNAs (miRNAs), which have long been known to regulate development. Several miRNAs, including miR‐124, miR‐155, and miR‐414 have been shown to modulate the development of monocytes in the bone marrow. miR‐124 is a brain specific miRNA that is expressed in microglial cells. It has been proposed that miR‐124, by directly inhibiting C/EBPα and PU.1, prevents microglia from acquisition of a reactive phenotype (Ponomarev et al., 2011). A microglial miRNAs signature has been recently identified in mice and humans (Butovsky et al., 2014). New experiments are needed to elucidate the roles of this subset of miRNAs unique or highly expressed in microglial cell. Meanwhile, recent data stress the relevance of miRNAs in microglia biology and show that interfering with biogenesis of miRNAs results in spontaneous microglia activation and accumulation of DNA damage in postnatal microglia, though density is unaltered (Varol et al., 2017). Available evidence confirms that miRNAs are key regulators of development and function of microglia cells. However, much work remains to be done to understand their roles and complex mechanisms of action.

## MECHANISMS REGULATING IDENTITY AND PLASTICITY OF BRAIN MACROPHAGES

Tissue macrophages are extraordinarily versatile cells with remarkable functional and morphological diversity. In the case of microglia, functional diversity encompasses surveillance of the surrounding microenvironment, phagocytosis during tissue remodeling (e.g., synaptic pruning) and debris clearance, neuromodulation of neuronal circuitry, and orchestration of innate and adaptive immune responses (Gomez‐Nicola & Perry, 2015). There is growing evidence that tissue‐specific factors from local microenvironment dictate the functional states of developing and adult tissue‐resident macrophages (Amit et al., 2016; Glass & Natoli, 2016)

### External Cues Involved in Differentiation and Maintenance of Brain Macrophages

Environmental signals received from nutrients, growth factors, cytokines, and cell–cell interactions are integrated by specific signaling pathways to modulate cell differentiation, growth, maturation and ultimately, to enforce fate decisions. The development of microglia is regulated by factors such as CSF‐1, interleukin 34 (IL‐34), and transforming growth factor beta (TGF‐β), which exert their actions during the early and late stages of primitive haematopoiesis. These factors have been recently identified as primary components promoting survival of microglia *ex vivo* (Butovsky et al., 2014; Bohlen et al., 2017). Mice deficient in TGF‐β in the brain show an important reduction of microglial cells beginning at E14.5 (Butovsky et al., 2014). This reduction was associated to an increase in apoptosis of these cells suggesting a role of TGF‐β in microglia survival and maintenance *in vivo* (Butovsky et al., 2014). CSF‐1, IL‐34 and its receptor, CSF‐1R, are important regulators of the differentiation of most macrophage populations both during development and in adult mice (Prinz et al., 2017). During primitive haematopoiesis CSF‐1R is required for the development and differentiation of EMP into microglia (Dai et al., 2002; Ginhoux et al., 2010; Erblich et al., 2011). *Csf‐1r* knockout mice are not viable and show complete absence of tissue macrophages, including microglia (Dai et al., 2002; Ginhoux et al., 2010; Erblich et al., 2011). CSF‐1R ligands CSF‐1 and IL‐34, seem to play redundant roles on CSF‐1R‐dependent signalling in microglial cells and tissue macrophages (Ginhoux et al., 2010; Greter et al., 2012; Wang et al., 2012). Mice that harbor a spontaneous mutation in the *Csf‐1* locus, *Csf‐1^op/op^* mice (Yoshida et al., 1990), leading to CSF‐1 deficiency, show altered morphology and mild reduction in the number of microglial cells, similarly to other tissue‐resident macrophages (Wegiel et al., 1998; Ginhoux et al., 2010). On the other hand, IL‐34 deficiency causes a approximately 40% reduction of microglial numbers, displaying certain degree of anatomical variability (Greter et al., 2012; Wang et al., 2012). Evidence also indicates that DAP‐12, a CSF‐1R and TREM‐2 adaptor protein, plays a role in microgliogenesis (Nataf et al., 2005; Otero et al., 2009). Both DAP‐12 and TREM‐2 are expressed in microglia, as well as immature dendritic cells and osteoclasts (Colonna, 2003). DAP‐12‐deficient mice show delayed post‐natal differentiation and migration of microglia into the CNS, whereas adult microglia show normal densities (Nataf et al., 2005). In contrast to these data, a study using a different *Dap‐12* knockout mouse line has reported reduced density of microglial cells in the basal ganglia and spinal cord of aged (10‐month‐old) mice (Otero et al., 2009). In humans, recessive mutations in DAP‐12 or its associated cell‐surface receptor TREM‐2 cause Nasu‐Hakola Disease, which is characterized by frontal dementia and bone cysts (Paloneva et al., 2000, 2002). Collectively, these data represent solid evidence of the important role of these growth factors in the regulation of differentiation and maintenance of microglia. However, the downstream mechanisms by which these endogenous differentiation factors exert their effects and whether they differentially influence the development and function of the distinct types of brain macrophages remains to be elucidated.

### Macrophage‐Specific Response to Environmental Signals Depends on the Activation of Developmentally Primed Enhancers

Macrophages are extremely plastic cells that quickly adapt their transcriptional outcome in response to an alteration in environment. Further, the same stimulus can trigger the activation of the same signaling pathway, including the same signal‐dependent transcription factor (e.g., NF‐κB), but different transcriptional response in different cells. To a large extent, this ability of the cells to activate a stimulus‐regulated transcriptional program in a cell type‐specific manner is achieved by cell type‐specific selection of the *cis*‐regulatory elements during acquisition of a differentiated cell fate (Ghisletti et al., 2010; Heinz et al., 2010, 2013). Evidence show that in tissue‐macrophages, there is a significant enrichment of DNA‐binding motifs for signal‐regulated transcription factors (e.g., Liver X receptors [LXRs]) in the vicinity of PU.1 bound sites. Moreover, presence of PU.1 was found to be required for LXR and TLR‐ (Toll‐like receptor) dependent gene expression(Heinz et al., 2010, 2013). These findings were consistent with those observed in bone marrow macrophages, in which TLR4 triggered recruitment of p300 to genomic locations exhibiting H3K4me1 (Ghisletti et al., 2010). Collectively, these data indicate that enhancer selection during development includes signal‐regulated enhancers that are primed for later activation in response to environmental input during adulthood, providing a mechanism driving stimulus‐ and cell type‐specific transcriptional response (Heinz et al., 2015; Link et al., 2015; Romanoski et al., 2015). There is very little work investigating remodeling of enhancer landscape during microglial activation. A very recent study has addressed this question in the context of the immune response in the spinal cord after sciatic nerve ligation, a model of neuropathic pain. This study analyses H3K4me1 chromatin mark in freshly isolated microglial cells in control conditions and several days after nerve injury to identify persistent changes driving neuropathic pain. The authors reported a sustained alteration in the levels of H3K4me1 in a restricted subset of enhancers (Denk et al., 2016). However, the choice of H3K4me1 may limit their ability to identify the extent of reorganization of the genomic landscape of *cis*‐regulatory elements after nerve injury. As discussed earlier, H3K4me1 is an epigenetic mark present, in conjunction with PU.1, at primed enhancers that are, in this way, bookmarked for a rapid activation, through recruitment of signal‐dependent transcription factors and deposition of H3K27ac, in response to environmental stimulus. In fact, only a small but functionally significant, proportion of *de novo* generated enhancers (latent enhancers) have been identified in macrophages in response to stimulus (Kaikkonen et al., 2013; Ostuni et al., 2013). Our knowledge about the genomic reorganizations driving inflammatory response of the brain innate immune cells remains very limited. Moreover, it comes primarily from other tissue‐resident macrophages outside the CNS.

### Local Microenvironment Shapes Brain‐Resident Macrophage Identity and Plasticity

Growing evidence strongly indicates that environmental factors shape brain macrophages during development and at steady‐state. In the adult, microglia tile the entire brain and have traditionally held to be a largely homogenous population that serve the same roles in all brain regions. However, recent data have challenge this view and instead reveal evidence suggesting that microglia represent a population of complex and functionally diverse cells. These studies show that during postnatal development and throughout adulthood region‐specific phenotypes of microglia emerge and require local cues to be maintained. Microglia anatomical, lysosome content, membrane properties, and transcriptome profile differ significantly across brain areas (Grabert et al., 2016; De Biase et al., 2017). Interestingly, local microglia transcriptional signatures are re‐established upon genetic or pharmacological ablation in the adult brain, indicating that local microenvironment continuously instructs the identity of microglia (De Biase et al., 2017). In line with these results, it was previously shown that external signals impinge on the enhancer landscape and gene expression profile of the local population of tissue‐resident macrophages (Gosselin et al., 2014; Lavin et al., 2014). Lavin and collaborators have shown that the environment is capable of reprograming differentiated macrophages when transferred into a new microenvironment (Lavin et al., 2014). The authors assessed the chromatin state of macrophages derived from transplanted adult bone marrow that replace embryo‐derived tissue‐resident macrophages upon lethal irradiation. They found that 4 months after engraftment, the donor transplant‐derived lung, spleen, liver, and peritoneal macrophages acquire enhancers found in embryonic macrophages in a tissue‐specific manner (Lavin et al., 2014). Further, it has recently been shown that environmental perturbations in specific developmental stages, such as those affecting the microbiome or prenatal immune activation, result in impaired microglia development and alterations in the associated transcriptional profile (Erny et al., 2015; Matcovitch‐Natan et al., 2016). Finally, expression‐profiling comparison demonstrates drastic differences between microglia isolated immediately *ex vivo* and *in vitro* cultured primary microglia (Butovsky et al., 2014). In line with these findings, transition of human and mouse microglia from the brain to tissue culture promotes remodeling of tissue‐specific enhancer landscape and extensive down‐regulation of genes that are induced in primitive mouse macrophages following migration into the fetal brain, consistent with their induction by local environmental factors (Gosselin et al., 2017). Moreover, another study has revealed that the loss of adult microglia‐specific transcriptional signature upon isolation and *in vitro* culture is reversed by engraftment of the primary cells back into an intact brain parenchyma (Bohlen et al., 2017). Although *in vitro* techniques and methodologies to culture tissue‐resident macrophages are under continuous development and improvement (Butovsky et al., 2014; Bohlen et al., 2017), data above pose notable questions on the utility of primary cultures of tissue‐resident macrophages and cell lines and urge caution when comparing and interpreting results obtained from *in vitro* experimental systems. These studies, taken together, highlight the importance of the microenvironment and constitutes an important body of evidence showing that microglia require sustained interaction with environmental cues to maintain their phenotypic identity and plasticity. However, the molecular mechanisms and the nature of tissue‐specific external signals remain largely unknown. It is very likely that the tight interaction observed between microglia and its local microenvironment also occurs in the case of non‐parenchymal macrophages. Whether environmental cues also shape the phenotype and functions of non‐parechymal macrophages of the brain is an exciting question that awaits further investigation.

## MACROPHAGE POPULATION DYNAMICS IN THE ADULT BRAIN

**Figure 3:**
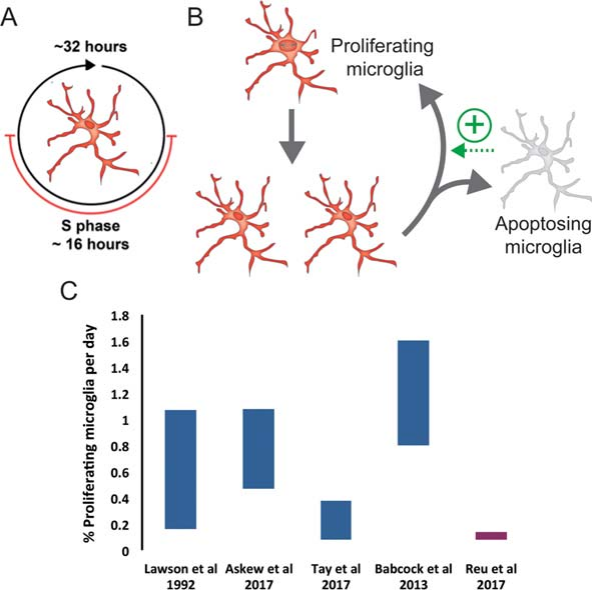
Cell cycle and proliferation rates of microglia. (A) Calculations of microglial turnover estimate a cell cycle length of approximately 32 h with S phase lasting approximately 16 h (Askew et al., 2017). (B) In the adult brain, the microglial population is maintained by self‐renewal of resident cells with no contribution from peripheral bone marrow‐derived cells. There is a tight temporal and spatial coupling between proliferation and apoptosis in order to maintain a stable cell density throughout lifetime, leading to a constant reorganization of the microglial landscape. (C) Comparison of the microglial proliferation rate (%) 24 h post‐labelling from studies in mice (blue) by Lawson et al. (1992), Babcock et al., (2013), Askew et al. (2017), Tay et al. (2017), and in human (red) by Reu et al. (2017). Note Lawson et al. used 3H‐thymidine and estimated a 0.05% proliferation rate by analyzing 2 h after dosing, instead of the 24 h shown here. Askew et al. used a single dose of BrdU in 24 h, whilst Tay et al. used cumulative BrdU (1 dose/d) and Babcock et al., used three doses of BrdU in 24 h (therefore allowing labeling of 1–2 S phases). Reu et al. used IdU and ^14^C. [Color figure can be viewed at http://wileyonlinelibrary.com]

A solid body of independent evidence overwhelmingly supports that microglial embryonic progenitors derive from the embryonic yolk sac, as described earlier (Ginhoux et al., 2010; Kierdorf et al., 2013a). However, for many years there was a strong debate focused on the peripheral versus central origin of microglia during embryonic development and in the adult brain. This long‐standing controversy was finally settled when a series of papers demonstrated that only when the blood brain barrier (BBB) is open, such as during irradiation and bone marrow transplantation, circulating monocytes can be found in the brain parenchyma (reviewed in Ginhoux et al., 2013; Prinz et al. 2017). Similarly, in several pathological models microglia‐like cells originate from bone marrow derived precursors (Flugel et al., 2001; Mildner et al., 2007; Varvel et al., 2012). In contrast, more refined experimental approaches using parabiosis, to create chimeric mice with shared circulation, demonstrated a constant infusion of monocytes into peripheral organs such as the spleen and the liver, but not into the brain parenchyma under physiological conditions (Ajami et al., 2007). This evidence was later supported by lineage‐tracing studies using different inducible mouse models in which a fluorescent reporter originally expressed by yolk sac embryonic progenitors (CX3CR1^+^, CSF1R^+^, Runx1^+^, Tie2^+^) was later found in daughter microglial cells (Ginhoux et al., 2010; Kierdorf et al., 2013a; Gomez Perdiguero et al., 2015; Hoeffel et al., 2015). Once the parenchymal origin of adult microglia was established it was just a matter of time to start the hunt for the mechanism maintaining their population. This investigation was also fostered by evidence indicating that adult microglia are also capable of recover their whole population after chemical or genetic depletion, prompting to suggest the existence of a microglial progenitor (Askew et al., 2017; Bruttger et al., 2015; Elmore et al., 2014). In the next sections, we will discuss the current knowledge of the dynamics of the microglial population in the healthy brain (Fig. 3).

### Cell Cycle and Proliferation Rates of Microglia

Maintenance of the microglial population by slow turnover of long‐lived cells has been assumed for many years, since Lawson *et al*. (1992) reported the turnover of mouse microglia using 3H‐thymidine labeling combined with F4/80 immunohistochemistry. The authors observed a substantial increase in the number of double‐labelled (3H‐thymidine+ F4/80+) cells between the 1‐ and 24‐h time points, with a lower number of double‐labelled cells between 24 and 48 h. It was hypothesized by the authors that peripheral proliferating macrophages were also labelled with 3H‐thymidine prior to infiltration into the CNS and differentiation into microglia, thus contributing to population turnover. However, this seems improbable given the current model of microglial origin and the unlikelihood of peripheral cells infiltrating the parenchyma and differentiating into microglia under physiological conditions. The authors justified selection of these time points to detect resident cells that underwent division within the parenchyma (1 and 24 h) or infiltrating cells that underwent division in the bone marrow prior to migration and differentiation (24 and 48 h). It was assumed that infiltrating cells would contribute to the population of proliferating cells in the later time points, thus the total number of proliferating resident microglia is likely to be underestimated by this study. Therefore, this data can now be re‐interpreted under a different light. It appears that there is a peak in microglial proliferation at 24 h post‐labeling, with 0.19% of microglial cells shown to be proliferating (Lawson et al., 1992). The reduction in 3H‐thymidine labelled cells between 24 and 48 h suggests that a large proportion of microglial proliferation occurs in the first 24 h after labeling (Lawson et al., 1992). Lawson and colleagues recognized the limitations of 3H‐thymidine (Rakic & Sidman, 1970), which may lead to underestimation of the total number of proliferating cells. Therefore, the understanding of the turnover of microglia was incomplete and needed re‐evaluation.

This turnover rate has been recently revisited with state‐of‐the‐art methods. Indeed, pulse‐and‐chase labeling with a single injection of the thymidine analog 5‐bromo‐2'‐deoxyuridine (BrdU), which incorporates in the nascent DNA during the SA phase, has led to the estimation of 0.7% proliferating microglia in the mouse brain under steady‐state conditions (Askew et al., 2017). Proliferation rates in the mouse brain assessed using labeling with Ki67, a commonly used proliferation marker, were in the same range but to some extent lower in a separate study (0.3–0.4%) (Tay et al., 2017), in spite of the fact that Ki67 labels cells in all active phases of the cell cycle and thus leads to higher estimation of proliferating cells compared with single BrdU labeling (Kee et al., 2002).

Another approach to analyze microglial population dynamics is based on chronic two‐photon imaging to assess cell duplications. Using γ‐retroviral vectors, which infect only dividing cells, and daily imaging over a 10–22‐day period led to the estimation of a microglial proliferation rate of 0.8% (Askew et al., 2017). However, another two‐photon study using inducible sparse tagging and tracking of individual microglia over prolonged periods of time, thanks to low dosing of tamoxifen, suggested instead an extremely low turnover rate (Fugger et al., 2017). By imaging the same cells every 2 weeks (until 6 months of age) or every month (from 6 months of age), the authors estimated the a proliferation rate of 0.13%. The discrepancy between these studies may arise from the imaging schedule, as the 2‐week approach from Fulger et al. may miss several cycles of division and lead to an underestimation of the microglial turnover.

Although these discrepancies produced different estimations of the number of average rounds of division in each microglia over the lifetime of mice and humans, as will be discussed in the next section, they both indeed demonstrate that under physiological conditions the microglial population undergoes active self‐renewal at low rates. In the human brain, microglial proliferation rates are similarly low. Gomez‐Nicola's group reported a 2% proliferation rate based on Ki67 staining (Askew et al., 2017), whereas Frisen's group reported microglial proliferation rates of 0.14% using iodo‐deoxyuridine labeling and 0.08% using retrospective C^14^ labeling in microglia purified by magnetic‐activated bead sorting (Reu et al., 2017). Importantly, the proliferation rate of microglia is around 100 times lower than for peripheral immune cells, such as granulocytes or monocytes (Reu et al., 2017). Although these studies show some discrepancies in the absolute turnover rates of microglia, they agree on a model of active and slow microglial turnover, which enables few cycles of renewal of the whole microglial population in a lifetime. It will be useful to see these studies on microglial turnover replicated with alternative approaches

In addition, further studies are also needed to understand the cell cycle dynamics of microglia, as the duration of the S phase during mitosis is directly related to the probability of detection of proliferating cells. Indeed, the shorter the S phase, the fewer cells would be labelled with BrdU thus resulting in an underestimation of the microglial cells undergoing mitosis. Estimations of the cell cycle using a BrdU time course have suggested that the S phase probably lasts around 16 h under steady‐state conditions (Askew et al., 2017). As in most cell types the S phase encompasses 50% of the whole cycle, it was estimated that microglia would undergo mitosis in approximately 32 h (Askew et al., 2017). This would be in agreement with reported cell cycle lengths of macrophages, which vary depending on differentiation stage from 20 to 40 h (Kueh et al., 2013). Similarly, a recent study that analyzed microglial proliferative response after facial nerve axotomy showed a seven 7‐fold increase in the number of cells from Day 2 to 7, which was compatible with approximately 3 divisions in 5 days. From these data a cell cycle of roughly 40 h during facial nerve axotomy (Tay et al., 2017), similar to the 32‐h cell cycle estimated in steady state conditions (Askew et al., 2017). However, more precise methods using double labeling with different halogenated forms of deoxyuridine such as chloro‐dU and iodo‐dU (Encinas et al., 2011), or cytometric analysis of DNA content using dyes such as Hoechst (Kim & Sederstrom, 2015) would help to precisely estimate the duration of the different phases of mitosis of microglia in health and disease. Overall, it would be of interest to import concepts and techniques from the stem cell field into future studies on microglial turnover.

Importantly, the proliferation of microglia is spatially and temporally coupled with microglial apoptosis, in order to keep constant their density (Askew et al., 2017). Many of the newborn microglia seem to have a short life time, as they have a higher probability of death in the first 5 days of cell life as determined by chronic two‐photon imaging (Askew et al., 2017). In agreement, the rate of new microglia appearing and disapearing were very similar in chronic two‐photon imaging experiments (Fuggert et al., 2017) and very few cell duplications are observed in the long term (36 weeks) after multicolor genetic lineage tracing using Microfetti mice, in which Confetti labeling is under the control of the CX3CR1 promoter (Tay et al., 2017). Similarly, the early study by Lawson *et al*. observed roughly equal numbers of labelled proliferating microglia and apoptotic (pycknotic) microglia, supporting the notion that many newborn microglia undergo cell death. Interestingly, newborn neurons too have similar high death rates in the first few days of life, both during embryonic development (de la Rosa & de Pablo, 2000) and in adult neurogenic niches (Sierra et al., 2010). However, we have little understanding of the molecular determinants of the coupling of microglial apoptosis and proliferation, and how these could provide a substrate for chronic dysregulation of the microglial population.

### Is Microglial Turnover Maintained by Progenitors?

The idea of the existence of a nestin^+^ microglial progenitor cell residing in the adult brain has been recently put forward based on repopulation studies. Nestin is an intermediate filament that is characteristically expressed in embryonic and adult neural stem cells and whose function has been related to their self‐renewal (Bellucci et al., 2011). Nonetheless, nestin has a wider expression in embryonic skeletal and cardiac muscle, liver, and kidney among other tissues (Neradil & Veselska, 2015). It seems to be particularly linked to mitosis, as is expressed by several proliferative cell types, from reactive astrocytes (Sierra et al., 2015) to vascular endothelial cells (Suzuki et al., 2010). Importantly, nestin is also considered a marker of cancer stem cells from neuroepithelial, epithelial, mesenchimal, and even germ cell origin, and it is used as a prognosis marker for cancer progression (Neradil & Veselska, 2015). In the steady‐state, nestin is expressed by neural stem cells (Lendahl et al., 1990), pericytes (Dore‐Duffy et al., 2006), and NG2^+^ oligodendrocyte precursor cells (Walker et al., 2010) within the CNS, with expression rarely found in other cell types (Gilyarov, 2008).

The first evidence of nestin expression in microglial progenitors came from Kim Green's group (Elmore et al., 2014), who observed a rapid repopulation of microglia following an almost complete depletion in adult mice after oral administration of high doses of PLX3397 (Pexidartinib), a potent inhibitor of receptor tyrosine kinases CSF‐1R, Kit, and Flt3. This reduced microglial numbers in the healthy brain by >90% after 7 days of treatment and almost completely depleted the population following longer treatment regimens (Elmore et al., 2014). In this model, the microglial population began to reconstitute 3 days after stopping treatment; however, repopulating cells displayed different morphology and expression of cell markers from control microglia, including transient expression of CD34 and c‐Kit, markers of HSCs, and nestin. By 14 days of recovery, microglial numbers, phenotype and morphology were indistinguishable from those in control brains (Elmore et al., 2014). The authors indirectly suggested the existence of an independent progenitor cell, which would be expected to undergo an asymmetric division to generate one single daughter microglia cell. In these experiments, a four‐fold increase in microglia was observed just 24 h after the depletion drug (pexidartinib) (Elmore et al., 2014). As it would have implied two full mitosis in 24 h (i.e., whole cell cycle of 12 h), the authors concluded that the repopulated cells must have come from an independent, unidentified cell type which after three days expressed nestin (Elmore et al., 2014). To further support the existence of a hidden progenitor, Elmore et al., performed a BrdU pulse‐and‐chase labeling experiment two days after the depletion. In an early time point (5 h), they observed that only 30% of the proliferating cells were microglia, whereas at later time points (24 h) virtually all proliferating cells were microglia, leading them to suggest that they must have originate from the remaining 70%, which was not characterized. There are however alternative interpretations. Indeed if absolute numbers of cells are used to analyze the transfer of the BrdU load, the data shows a larger number of BrdU microglia at 5 h than at 24 h, making unnecessary the existence of an independent progenitor to justify the number of BrdU microglia at 24 h. In addition, the decrease observed between 5 and 24 h could result from dilution of the BrdU label, which cannot be assessed because the BrdU concentration was not reported (Elmore et al., 2014); or from early death of the proliferating cells, as has indeed been shown by two‐photon imaging (Askew et al., 2017).

Similar results were obtained after transgenic ablation of microglia: the microglial population is rapidly reconstituted by proliferation of resident cells. The use of a *CX3CR1* ^CreEr^‐based system crossed with an inducible diphtheria toxin receptor system allowed specific ablation of microglial cells following tamoxifen and diphtheria toxin injections (Bruttger et al., 2015). An 80% reduction in microglia was observed 3 days after induction, but after 14 days cell numbers returned to baseline levels. In agreement with the pharmacological depletion model, after the transgenic ablation of microglia pools of resident cells, which rapidly proliferate to colonize the parenchyma, rapidly reconstituted the population. These CNS‐derived microglia also expressed nestin; however, this expression was transient and has no longer seen once cell numbers returned to normal at Day 14.

In contrast, nestin expression has not been observed in proliferating microglia in steady‐state conditions using nestin‐GFP mice (Askew et al., 2017). It is important to note that the fluorescent reporter has a long life in this mouse strain (longer than the actual nestin protein), leading to its detection in daughter cells originated from the nestin^+^ neural stem cells (Encinas & Enikolopov, 2008), therefore increasing the probability of detection of nestin in microglial progenitors. In addition, lineage tracing of nestin‐expressing neuroprogenitor cells has never been reported to produce microglial cells, at least in the hippocampus, neither at baseline or disease conditions (Encinas et al., 2011, Sierra et al., 2015). Therefore, the putative expression of nestin, a typical ectodermal marker, in microglial progenitors and whether its expression is exclusively related to their proliferative response, to depletion, or to more physiologically relevant paradigms remains to be determined. With these ideas in mind, it is possible that expression of nestin on microglia is induced only following disruption of population homeostasis, as microglial expression of nestin under physiological conditions has not yet been reported (Hickman et al., 2013; Butovsky et al., 2014; Grabert et al., 2016). Furthermore, the cytokine storm observed by Bruttger *et al*. highlights how far from a physiological system these paradigms are, as the inflammatory milieu of the CNS is markedly affected following massive microglial cell death. Therefore, these data suggest that nestin expression in microglia reflects a transient repopulating phenotype, rather than a subpopulation of “microglial progenitor cells” (Waisman et al., 2015).

Regardless of the nestin expression, the key question underlying these studies is whether every microglia has the capacity to self‐renew or there is a microglial progenitor cell. Direct evidence of microglia self‐renewing in steady state conditions was obtained using chronic two‐photon imaging, during which microglia were observed to proliferate and give rise to two identical daughter cells, which became ramified and distanced from each other over a 3‐week period (Askew et al., 2017). These results suggest that at least in physiological conditions all microglia have the ability to self‐renew, undergoing symmetric divisions to give rise to identical daughter (microglial) cells. Similar conclusions were reached using Microfetti mice, where computational Montercalo simulations supported a model where every microglia had the ability to self‐renew, instead of microglial turnover being dependent on microglial progenitors (Tay et al., 2017). Similarly, the authors observed that following axotomy evenly distributed clusters of two to four daughter microglial cells were generated, suggesting that clonal expansion of microglia and not a specialized microglial progenitor was sufficient to produce the increased number of cells after acute injury (Tay et al., 2017). Finally, mathematical modeling of microglial turnover in the human brain using retrograde ^14^C labeling was consistent with a homogeneous proliferation of microglia but not with the proliferation of a subpopulation of quiescent, long‐lived progenitor cells (Reu et al., 2017).

In summary, the quest for the microglial progenitors remains on. Although direct evidences using two‐photon imaging and lineage tracing analysis strongly support that every microglia cell has the capacity to self‐renew, the intriguing possibility that a yet unidentified cell type which would act as a progenitor remains to be more directly examined in terms of marker expression, location, cell cycle dynamics, and recruitment under different physiological and pathological conditions.

## THE ADDITIONAL BRAIN INNATE IMMUNE TOOLKIT: PERIVASCULAR, MENINGEAL, AND CHOROID PLEXUS MACROPHAGES

Although it is common to refer to microglia as “the brain immune cells”, there are indeed several other populations of resident macrophages located in strategic barrier regions: the perivascular space, the meninges, and the choroid plexus. Interestingly, the repertoire of immune cells residing in the brain's interfaces is much complex than previously assumed, as recently defined by Korin et al. (2017) using CyTOF mass cytometry. The naïve brain contains subsets of CD8 T cells, B cells, NK cells and dendritic cells, residing at the meninges, choroid plexus and parenchyma (Korin et al., 2017). For simplicity, in this section we will focus on the populations of brain macrophages. Early papers used irradiation and bone marrow chimeras to search for the brain antigen presenting cells (APCs), that is, the cells that in conditions such as multiple sclerosis would be responsible for interacting with lymphocytes to activate the adaptive branch of the immune response. These papers suggested that brain APCs were located in the perivascular space and originated from the bone marrow (Hickey & Kimura, 1988; de Groot et al., 1992). However, as discussed earlier, irradiation leads to inflammation, opening the BBB, and leading to the leaking of circulating monocytes with bone marrow origin (Kierdorf et al., 2013b). An improved bone marrow transplantation method was developed to reduce inflammation, where mice received low doses of irradiation and both syngenic hematopoietic and mesenchymal stem cells are injected intra‐bone marrow (IBM) (Hasegawa‐Ishii et al., 2013). Using this method, bone‐marrow GFP‐labelled cells were found in the leptomeninges and choroid plexus as early as 2 weeks, followed by clusters of bone marrow cells in small but growing numbers from 1 to 8 months in discrete areas of the brain parenchyma, such as habenula, amygdala, hippocampus, and cerebellum. As these regions are very close to choroid plexuses, the authors suggested a novel entry point to the brain parenchyma of bone marrow cells followed by local expansion by proliferation (Hasegawa‐Ishii et al., 2013). The replenishment of choroid plexus macrophages from circulating cells has also been demonstrated using genetic lineage tracing and parabiosis (Goldmann et al., 2016). However, the IBM study did not directly assessed BBB opening or inflammation (Hasegawa‐Ishii et al., 2013). The lack of differentiation of bone marrow cells into parenchymal microglia under non‐inflammatory conditions is strongly sustained by the literature (Ginhoux et al., 2010; Kierdorf et al., 2013a; Gomez Perdiguero et al., 2015; Hoeffel et al., 2015) but nonetheless the provocative hypothesis that in some circumstances circulating precursors may enter the parenchyma through the choroid plexus deserves further testing. Furthermore, more experiments are required to determine the rate of replacement of choroid plexus macrophages from bone marrow cells, their half‐life, and their differentiation program.

In contrast to choroid plexus macrophages, perivascular and meningeal macrophages originate from the embryonic yolk sac just like microglia, as compellingly demonstrated by parabiosis and lineage tracing of CX3CR1 expressing cells at P9 up to 10 months (Goldmann et al., 2016). Perivascular macrophages in the spinal cord are maintained by self‐renewal even during inflammation induced by autoimmunity with myelin‐derived peptides (Goldmann et al., 2016). Following research on microglia, further studies will determine whether these cells retain proliferative properties and renew their population over the lifetime.

### Functions of Perivascular, Meningeal, and Choroidal Macrophages

These sets of resident macrophages have been largely disregarded into oblivion, but recent research has shed light into their relevant roles in the perivascular space, meninges, and choroid plexus (Herz et al., 2017). A key feature is their location at the so‐called “brain interfaces”, which act as communicating bridges between the brain parenchyma and the outside.

The best known barrier, the BBB, is formed between the blood and the brain parenchyma. The BBB is a highly selective barrier formed by tight junctions on the endothelial cells of the brain capillaries; a perivascular space separating the basal membrane of these capillaries where both pericytes and perivascular macrophages reside; and the terminal feet of parenchymal astrocytes (Daneman, 2012). Brain vascularization begins during early embryonic development at E11 in mice and is followed by the recruitment of pericytes, which are essential for the establishment of the BBB, at around E13.5 (Daneman et al., 2010). Pericytes were long though to derive from either neural crest cells or mesenchymal cells in the blood vessels although it has been suggested that a subpopulation derives from differentiated macrophages expressing F4/80 and CD31 during initial brain vascularization (Yamamoto et al., 2017). These events are followed by the recruitment of yolk sac precursors from E9 to E16 to form perivascular macrophages, although the precise timing remains to be determined (Goldmann et al., 2016). Finally, astrocytes are recruited to the BBB 1 week after birth (Daneman et al., 2010).

The roles of perivascular macrophages have been poorly studied. These elongated cells are very similar to microglia in their expression of markers such as Iba1, CD11b, Csf1R (CSF receptor 1), and CX3CR1 (Goldmann et al., 2016; Sierra et al., 2007). Perivascular macrophages had a very similar transcriptional profile compared with microglia, but clearly different from circulating monocytes (Goldmann et al., 2016). In the adult brain, perivascular macrophages are presumed to be involved in immune surveillance (Daneman, 2012), possibly related to their continuous retraction and protraction of processes along blood vessels (Goldmann et al., 2016). They play a protective role during bacterial infection by regulating the recruitment of circulating leukocytes (Polfliet et al., 2001), although their role during neurodegenerative conditions in which BBB is altered remains to be determined (Daneman, 2012). Interestingly, developmental defects in CSF1R signaling lead to defective pericyte coverage of brain vessels (Yamamoto et al., 2017), suggesting a role of perivascular macrophages in BBB establishment.

The BBB is also closely related to the meninges, which encompass three layers of connective tissue: the external dura, facing the skull; and the arachnoid and pia (leptomeninges), separated by the subarachnoid space infused with cerebrospinal fluid, and through which blood vessels penetrate the brain parenchyma (Coles et al., 2017). Leptomeningeal epithelial cells associate to large blood vessels only, at least in the human brain (Coles et al., 2017). Meninges are established in the early embryonic development around E9–E10 in the mouse brain. Dural cells derive from the mesoderm and are related to the skull bones, whereas leptomeningeal cells derive from the neural crest (Siegenthaler & Pleasure, 2011; Goldmann et al., 2016). Importantly, it was recently discovered that meninges possess a system of lymphatic vessels whose structure is largely similar to blood vessels, comprising endothelial cells, but through which interstitial fluid and immune cells (lymph) flow towards deep cervical lymph nodes (Louveau et al., 2015). This so‐called “glymphatic” system connects the cerebrospinal fluid with the peripheral immune system and has been suggested to play a role in the clearance of brain waste products, such as βAmyloid (Raper et al., 2016). Importantly, little is known about meningeal macrophages. Resident macrophages originated in the yolk sac during embryonic development with ameboid morphology and motility have been described in the mouse meninges (Goldmann et al., 2016), but their particular location in the dura and/or leptomeninges is unknown, possibly due to the reduced size of mouse meninges (Raper et al., 2016). Meningeal macrophages have also been observed in human meninges, but whether they are part of the glympathic system is unknown (Louveau et al., 2015). Interestingly, macrophages instruct lymphangiogenesis during development by regulating proliferation of lymphatic endothelial cells in the peripheral immune system (Gordon et al., 2010), raising the possibility that brain meningeal macrophages play similar roles. In addition, during autoimmune diseases such as experimental autoimmune encephalomyelitis, a mouse model of multiple sclerosis, the meninges are an important site of antigen presentation to T cells (CD4+), performed both by resident macrophages and dendritic cells (Kivisakk et al., 2009). Yet, many fundamental questions about the location and function of meningeal macrophages under steady state and pathological conditions remain unanswered.

A second barrier is formed between blood and cerebrospinal fluid and is sustained by tight junctions between epithelial cells in the choroid plexus (Engelhardt & Sorokin, 2009). Choroid plexus are evolutionarily conserved, highly vascularized structures that protrude into the brain ventricles as well as the subarachnoid space (Bill & Korzh, 2014). Choroid plexus are made of epithelial cells specified from the neuroectoderm as early as E10.5 in the mouse embryo (Liddelow, 2015). In addition, the choroidal stroma includes several cell types such as fibroblasts, neural stem cells, telocytes (a novel type of intersticial cell with long and thin processes and regenerative properties), as well as circulating lymphocytes and resident macrophages (Bill & Korzh, 2014).These macrophages are initially seeded from the yolk sac during embryonic development (E9.5–E16 in mice) but are constantly replaced by bone marrow derived monocytes throughout adulthood (Goldmann et al., 2016). The main functions of the choroid plexus (i.e., barrier maintenance and cerebrospinal fluid release) are carried out by epithelial cells. The cerebrospinal fluid contains many bioactive molecules whose significance has just begun to be unraveled but seem to be involved in autonomic functions such as sleep and appetite, neural transmission, brain development, and a growing list of neurodegenerative conditions, in addition to providing a hydrostatic skeleton that absorbs mechanical brain damage (Bill & Korzh, 2014). The role of choroid plexus macrophages has not been fully resolved, but they are located close to the microvilli of the choroid plexus (Goldmann et al., 2016) and would be interesting to assess their contribution to cerebrospinal fluid release and flux. In addition, choroid plexus are to some extent related to circumventricular organs (CVOs), which include secretory (neurohypophysis, pineal gland, median eminence, subcommisural organ) and sensory (area postrema, subfornical organ, organum vasculosum of the lamina terminalis) CVOs (Kaur & Ling, 2017). CVOs, separated from the brain parenchyma by modified ependymal cells named tanicytes (Kaur & Ling, 2017), are highly vascularized with fenestrated capillaries and located adjacent to the ventricles, and their main function is to release substances into the cerebrospinal fluid (Bill & Korzh, 2014). All CVOs contain macrophages (Kaur & Ling, 2017), whose origin and function remains to be discovered.

## CONCLUSION AND PERSPECTIVE

Investigation of the mechanisms governing microglia and brain macrophage development and function has come a long way. So far, likely because of their higher abundance, the vast majority of the studies have focused on microglia cells. The use of single‐cell RNA sequencing has allowed interrogating the gene expression profile of the different types of brain macrophages (Goldmann et al., 2016; Zeisel et al., 2015). In the future, the combination of emerging single‐cell epigenomic methods interrogating chromatin accessibility (scATAC‐seq), chromatin histone modifications (scChIP‐seq), and DNA methylation (scBS‐seq) coupled to single‐cell gene expression profiling (scRNA‐seq) should allow us to capture similarities and differences in gene expression and epigenetic chromatin landscape also in less abundant cell populations of non‐parenchymal brain macrophages (Smallwood et al., 2014; Buenrostro et al., 2015; Guo et al., 2015; Rotem et al., 2015; Angermueller et al., 2016; Clark et al., 2016). The recent development and widespread adoption of new genome editing techniques combined with single‐cell genomic approaches open new possibilities including forward genetic screens, which will undoubtedly contribute to uncover new players regulating microgliogenesis and development of non‐parenchymal macrophages.

Finally, the role of the different intrinsic factors and the nature of the external signals that regulate fate decisions and commitment to differentiation of progenitors to brain macrophages are not yet completely understood. Similarly, a variety of environmental factors whose identities remain to be identified, shape and maintain the identity of microglia and non‐parenchymal macrophages across the different brain areas. Many aspects of the mechanisms driving brain colonization and terminal differentiation processes which lead to the generation of distinct types of brain resident macrophages during early postnatal stages are only partially understood. Other questions as to how microglial cells communicate to each other and distribute within the adult brain to form a mosaic‐like network to scan the surrounding environment and modulate the neuronal circuitry underlying brain functions remains enigmatic. The vibrant and exciting field of immune cells of the brain and neuroinflammation has recently undergone a dramatic transformation. However, there are many fundamental questions that remain unanswered. A deeper understanding of the mechanisms that regulate development, maintenance of phenotypic identity, population dynamics, and function of resident macrophages of the adult brain should help clarifying how these cells react to brain disorders and will offer new targets to develop novel therapeutic interventions for these devastating conditions.
